# *Ex Vivo* Expansion of Murine MSC Impairs Transcription Factor-Induced Differentiation into Pancreatic *β*-Cells

**DOI:** 10.1155/2019/1395301

**Published:** 2019-03-10

**Authors:** Dario Gerace, Rosetta Martiniello-Wilks, Rosaline Habib, Binhai Ren, Najah Therese Nassif, Bronwyn Anne O'Brien, Ann Margaret Simpson

**Affiliations:** ^1^The School of Life Sciences and Centre for Health Technologies, Faculty of Science, University of Technology Sydney, Sydney, Australia; ^2^Translational Cancer Research Group, University of Technology Sydney, Sydney, Australia

## Abstract

Combinatorial gene and cell therapy as a means of generating surrogate *β*-cells has been investigated for the treatment of type 1 diabetes (T1D) for a number of years with varying success. One of the limitations of current cell therapies for T1D is the inability to generate sufficient quantities of functional transplantable insulin-producing cells. Due to their impressive immunomodulatory properties, in addition to their ease of expansion and genetic modification *ex vivo*, mesenchymal stem cells (MSCs) are an attractive alternative source of adult stem cells for regenerative medicine. To overcome the aforementioned limitation of current therapies, we assessed the utility of *ex vivo* expanded bone marrow-derived murine MSCs for their persistence in immune-competent and immune-deficient animal models and their ability to differentiate into surrogate *β*-cells. CD45^−^/Ly6^+^ murine MSCs were isolated from the bone marrow of nonobese diabetic (NOD) mice and nucleofected to express the bioluminescent protein, *Firefly luciferase (Luc2)*. The persistence of a subcutaneous (s.c.) transplant of *Luc2*-expressing MSCs was assessed in immune-competent (NOD) (*n* = 4) and immune-deficient (NOD/*Scid*) (*n* = 4) animal models of diabetes. *Luc2*-expressing MSCs persisted for 2 and 12 weeks, respectively, in NOD and NOD/*Scid* mice. *Ex vivo* expanded MSCs were transduced with the HMD lentiviral vector (MOI = 10) to express furin-cleavable human insulin (*INS-FUR*) and murine *NeuroD1* and *Pdx1*. This was followed by the characterization of pancreatic transdifferentiation via reverse transcriptase polymerase chain reaction (RT-PCR) and static and glucose-stimulated insulin secretion (GSIS). *INS-FUR*-expressing MSCs were assessed for their ability to reverse diabetes after transplantation into streptozotocin- (STZ-) diabetic NOD/*Scid* mice (*n* = 5). Transduced MSCs did not undergo pancreatic transdifferentiation, as determined by RT-PCR analyses, lacked glucose responsiveness, and upon transplantation did not reverse diabetes. The data suggest that *ex vivo* expanded MSCs lose their multipotent differentiation potential and may be more useful as gene therapy targets prior to expansion.

## 1. Introduction

T1D results from the autoimmune destruction of the pancreatic insulin-producing *β*-cells, which leads to hyperglycaemia and the lifelong dependence on exogenous insulin therapy [1]. Currently, the only cures for T1D are pancreas or islet transplantation; however, these interventions are limited by a shortage of donor organs and the requirement for lifelong immunosuppression [2]. To overcome the limitations of current therapies, a promising alternative strategy is the *ex vivo* generation of surrogate *β*-cells through the directed-differentiation of nonpancreatic target cells [3–10].

Pancreatic transcription factors play an important role both in islet cell differentiation and specialization and mature *β*-cell function during embryonic and neonatal development and adult life, respectively [11, 12]. Our laboratory and others have investigated the direct transfer of *β*-cell transcription factors and insulin as mediators of pancreatic transdifferentiation in nonpancreatic cells/tissues with varying success [3–5, 13–17]. We have previously shown that the endocrine-specifying transcription factor, *NeuroD1*, which lies downstream of *Pdx1* in the transcription factor hierarchy of pancreatic development, was capable of inducing pancreatic transdifferentiation of a rat hepatocyte cell line (H4IIE). Transdifferentiation was characterized by the upregulation of both upper and lower hierarchy pancreatic transcription factors, without the development of exocrine differentiation [12, 18]. In addition, due to the overexpression of the furin-cleavable human insulin (*INS-FUR*) gene (a modified form of human proinsulin, which permits cleavage into mature insulin via furin enzymes in nonpancreatic cells), these cells were capable of synthesizing, storing, and secreting mature human insulin in a glucose-responsive manner and reversed diabetes upon transplantation in STZ-diabetic NOD/*Scid* mice [18]. However, one of the current challenges of clinical translation of combinatorial gene and cell therapies for T1D is upscaling the production of functional surrogate *β*-cells [19].

Due to their high plasticity, immunomodulatory properties, fewer ethical concerns, and ease of *ex vivo* expansion and gene modification [20, 21], MSCs are an attractive alternative target cell for the autologous and allogeneic treatment of T1D. Several studies have investigated the *ex vivo* targeting of MSCs for transdifferentiation into islet progenitor cells (IPCs) via viral-mediated transfer of pancreatic transcription factors [14–17]. Previously, the transfer of the “master regulator” of pancreatic differentiation, *Pdx1*, to MSCs resulted in their differentiation into glucose-responsive IPCs, which reversed diabetes, for a period of 6-8 weeks (experimental endpoint), upon transplantation into STZ-diabetic NOD/*Scid* mice [14]. However, *Pdx1* transfer has also been associated with exocrine differentiation and concomitant tissue damage, which is undesirable for a T1D cell replacement therapy [22]. Therefore, in this study, we assessed the pancreatic differentiation potential of *ex vivo* expanded murine bone marrow-derived MSCs as a preclinical model to overcome the shortage limitations of current therapies, via the overexpression of murine *NeuroD1* and *INS-FUR* using a lentiviral vector. We found that due to a loss of the intrinsic multipotent differentiation potential of MSCs with increasing culture, transcription factor-mediated *β*-cell differentiation, via the forced expression of *NeuroD1* and *INS-FUR*, failed to occur. This was confirmed via the overexpression of murine *Pdx1*, which is known to induce *β*-cell differentiation of MSCs at early passage numbers. The data highlight the limited timeframe for MSCs to function as effective gene therapy targets and suggest that MSCs do not represent a suitable alternative source of cells to overcome the shortage limitations of current *β*-cell replacement therapies.

## 2. Methods

### 2.1. Sourcing of Animals

NOD and NOD/*Scid* mice were sourced from the Animal Resources Centre (WA, Australia). All animal work was approved by the UTS Animal Care and Ethics Committee (ACEC 2011-447A; ACEC 2009-244A) and complied with the Australian code for the care and use of animals for scientific purposes [23].

### 2.2. MSC Isolation and Cell Culture

Bone marrow was flushed from the femurs of female NOD mice (6-8weeks old), and the cell pellet was resuspended in standard MSC medium (*α*-minimal essential media (MEM), 1% v/v 100x Penicillin/Streptomycin/L-Glutamine (P/S/G) with 20% v/v Fetal Bovine Serum (FBS)) (Gibco^®^, Thermo Fisher) and incubated at 37°C/5% CO_2_. Plastic-adherent stromal cells were subcultured for two passages (with epiphyses) prior to fluorescence-activated cell sorting (FACS).

Passage 2 plastic-adherent stromal cells (5 × 10^5^ cells) were resuspended in sorting buffer (1x Hanks Balanced Salt Solution (HBSS) supplemented with 5% v/v FBS) and stained with 0.2 mg/ml rat anti-mouse CD45 monoclonal antibody (mAb) conjugated to allophycocyanin (APC) (BD Pharmingen™, USA) and 0.2 mg/ml rat anti-mouse Ly6 (Sca-1) mAb conjugated to phycoerythrin (PE) (BD Pharmingen™, USA). Stained stromal cells were sorted by FACS at the Advanced Cytometry Facility (Centenary Institute, Sydney, Australia) using a BD FACSAria™ II flow cytometer and analysed using BD FACSDiva™ software (Version 6.1.3). The stromal cells were sorted into CD45^−^/Ly6^+^ (MSCs) and CD45^+^/Ly6^+^ (double positive) cell populations. Sorted cells were resuspended in complete medium and incubated at 37°C/5% CO_2_. Following cell attachment, 10 ng/ml basic fibroblast growth factor (bFGF) was added to the standard MSC medium, in which the parental stromal cells and sorted cells were cultured thereafter.

### 2.3. MSC Proliferation and Clonogenicity

For proliferation assays, MSCs at early (P3-15), mid (P15-30), and late (P30-60) passage numbers were seeded in 24-well plates (2.5 × 10^3^ cells/well) (Falcon^®^ BD Biosciences, San Jose, USA) in triplicate and maintained in standard MSC medium for 15 days, with the medium replenished weekly. Cell viability was assessed by Trypan blue (0.4% v/v; Gibco^®^, Thermo Fisher) exclusion. Total cell and viable cell numbers were determined and represented as mean ± standard deviation (SD) for each time point (*n* = 3).

For clonogenicity assays, MSCs at early, mid, and late passage numbers were seeded in 10 cm^2^ tissue culture-treated plates (5 × 10^2^ cells/plate) (Falcon^®^ BD Biosciences) and maintained in standard MSC medium for 10 days. Colonies were stained with 0.4% v/v methylene blue in methanol and counted by microscopy. Data were represented as mean colony count per 5000 cells ± SD (*n* = 3). Standard MSC medium was replenished weekly.

### 2.4. Morphological Analysis

Images of four fields of view at 10x or 20x magnification were acquired at early, mid, and late passage numbers using a Leica^©^ DM microscope (Leica Microsystems^©^, Wetzlar, Germany) and processed using the image processing software, Leica Application Suite (V4.4.0) (Leica Microsystems^©^). Scale bars on figures are equivalent to 100 *μ*m.

### 2.5. Gene Expression Profiling

Total RNA was extracted using TRIzol^®^ Reagent (Thermo Fisher^®^, Waltman, USA) and samples were treated with DNase I, Amplification Grade (Thermo Fisher^®^, USA), before cDNA synthesis using SuperScript^®^ III First-Strand Synthesis SuperMix (Thermo Fisher^®^, USA). RT-PCR was subsequently performed using an Eppendorf^®^ Mastercycler (Eppendorf™, Hamburg, Germany) to determine the relative expression levels of selected pancreatic genes using GoTaq PCR Master Mix (Promega^®^, Madison, USA) and the previously published oligonucleotide sequences and optimised PCR protocols (Supplementary Table 1) [4]. PCR products were imaged after electrophoresis on a 1% w/v agarose gel stained with 10000x GelRed™ (Biotium^®^, Fremont, USA) (1 : 100000) on the InGenius3 (Syngene^®^, Frederick, USA) UV transilluminator using the GeneSys image acquisition software (Syngene^®^).

### 2.6. Differentiation Assays

#### 2.6.1. Adipogenesis

Early, mid, and late passage number cells were seeded in standard MSC medium in 24-well plates (2.5 × 10^4^ cells/well) in triplicate and grown to 80-90% confluence. The medium was subsequently replenished with either adipogenic control or differentiation medium, as previously described [24]. The cells were stained with 0.2% w/v Oil Red O in methanol (Fronine^®^, Sydney, Australia) and semiquantitatively scored as previously described [24]. Values were expressed as count per cm^2^ and were represented as means ± SDs (*n* = 3).

#### 2.6.2. Osteogenesis

Early, mid, and late passage cells were seeded in standard MSC medium in 24-well plates (1.25 × 10^4^ cells/well) in triplicate and grown to 90-95% confluence. The medium was subsequently replenished with either osteogenic control or differentiation medium, as previously described [24]. The cells were stained with 2% w/v Alizarin Red S (pH 4.1) (Fronine^®^) and semiquantitatively scored, as previously described [24]. Values were expressed as count per cm^2^ and were represented as means ± SDs (*n* = 3).

#### 2.6.3. Chondrogenesis

Early, mid, and late passage cells were seeded in 24-well plates (1.25 × 10^4^ cells/well) and grown to 90% confluence in standard MSC medium. The medium was subsequently replenished with either control (MesenCult™-ACF Chondrogenic Differentiation Basal Medium (STEMCELL Technologies^®^, Vancouver, Canada) with 2 mM L-glutamine) or differentiation (MesenCult™-ACF Chondrogenic Differentiation Basal Medium, 2 mM L-glutamine, MesenCult™-ACF 20X Chondrogenic Differentiation Supplement) medium and incubated at 37°C/5% CO_2_ for 18 days. On day 18, the cells were fixed in 10% v/v neutral buffered formalin and stained with Alcian blue solution (8x, pH 2.5) (Sigma-Aldrich™, Sydney, Australia). Chondrogenesis was visualised by Alcian blue staining of filamentous glycosaminoglycans.

### 2.7. Construction of Mammalian Plasmid pVITRO-*Luc2*

The manipulation of genetic material and generation of genetically modified organisms were approved by the UTS Biosafety Committee (2001-19-R-GC; 2009-02-R-GC). The luciferase reporter gene *Luc2* (*Photinus pyralis*), encoded within the vector pGL4.20 (*Luc2*/Puro) (Promega^®^, Ipswich, USA), was digested with the restriction enzymes, EcoRV-HF^®^ and BamHI-HF^®^ (New England Biolabs^®^, San Diego, USA), and ligated into the mammalian dual expression plasmid pVITRO2-hygro^®^-mcs (InvivoGen^®^, San Diego, USA) to generate the mammalian bioluminescence plasmid pVITRO2-*Luc2* (Supplementary Figure 1a).

### 2.8. Nucleofection

Early passage MSCs (1 × 10^6^ cells) were nucleofected with 5 *μ*g pVITRO2-*Luc2* and 2 *μ*g pmax-GFP^®^, according to the manufacturer's instructions (Lonza™, Basel, Switzerland), using the Nucleofector™ 2b device (Lonza™). Following nucleofection, the cells were returned to culture in standard MSC medium at 37°C/5% CO_2_ for one week. Stable clones were then selected using 200 *μ*g/ml Hygromycin B (Thermo Fisher Scientific^®^) over a two-week period.

### 2.9. *In Vitro* Bioluminescence Imaging (BLI)

*In vitro* BLI of a linear concentration of midpassage MSC-*Luc2* and the cell line MSC-*Luc2/LacZ ID7* (positive control) was performed in 96-well ViewPlate microplates (PerkinElmer^®^, Waltman, USA). Cells were attached overnight and imaged on the IVIS Lumina II (PerkinElmer^®^) following the addition of 150 *μ*g/ml D-luciferin (Gold Biotechnology^®^, St. Louis, USA). For quantification, a region of interest (ROI) was manually selected using the Living Image (Version 3.1) software. BLI intensity values were represented as the mean average radiance ± SD (p/s/cm^2^/sr).

### 2.10. *In Vivo* MSC Persistence

NOD (*n* = 4) and NOD/*Scid* (*n* = 4) mice (6-10 weeks of age) received a total of six subcutaneous (s.c.) injections of 1 × 10^4^ (*n* = 2), 1 × 10^5^ (*n* = 2), and 1 × 10^6^ (*n* = 2) midpassage MSC-*Luc2* cells/mouse. Untreated age-matched NOD (*n* = 2) and NOD/*Scid* (*n* = 2) mice were utilized as negative controls. Mice were anaesthetised using 2.5% isoflurane carried in O_2_ (1.5 l/min), transferred to the IVIS Lumina II imaging unit, and maintained under anaesthesia. BLI images were acquired 5 min after the intraperitoneal (i.p.) injection of D-luciferin (15 mg/ml) at 150 mg/kg or 10 *μ*l/g. For quantification, ROI was manually selected using the Living Image (Version 3.1) software. BLI intensity values were presented as the mean average radiance ± standard errors of means (SEMs) (p/s/cm^2^/sr).

### 2.11. Construction of Lentiviral Plasmids

The pHMD and pHMD-*INS-FUR* lentiviral plasmids [3–5] were modified to express *INS-FUR* and the human codon-optimized murine (*Mus musculus*) *Neurod1* and *Pdx1* genes. Using GeneArt Gene Synthesis™ (Thermo Fisher^®^, USA), murine *Neurod1* cDNA (NM_010894) was synthesized-linked to eGFP via a T2A peptide at the C-terminus. The *Neurod1-T2A-eGFP* sequence was PCR-amplified using the forward and reverse primers, 5′-GATACTTGGCCATATGACCAAATCATACAGCGA-3′ and 5′-CCATGAGGCCCAGTTAAT-3′, containing MscI and the PacI restriction sites, respectively. PCR-amplified *Neurod1-T2A-eGFP* was ligated into pHMD and pHMD-*INS-FUR* following digestion with MscI and PacI (New England Biolabs^®^, Waltman, USA) to generate pHMD-*Neurod1* and pHMD-*Neurod1/INS-FUR*, respectively.

The pAAV-*Pdx1* plasmid (donated by Dr. Grant Logan, Children's Medical Research Institute, Westmead Children's Hospital, Sydney, Australia) containing the murine *Pdx1* cDNA (NM_008814.3) and the fluorescent reporter mCherry upstream and downstream of the internal ribosomal entry site (IRES), respectively, was used to clone murine *Pdx1* into the HMD lentiviral plasmid. The *Pdx1-IRES-mCherry* sequence was PCR-amplified using the forward and reverse primers, 5′-GATACTGGATCCATGAACAGCGAGGAACAG-3′ and 5′-GCGCCGTTAATTAATTACTTGTACAGCTCGTC-3′, containing BamHI and PacI restriction sites, respectively. PCR-amplified *Pdx1-IRES-mCherry* was ligated into pHMD following digestion with BamHI-HF^®^ and PacI (New England Biolabs^®^, USA) to generate pHMD-*Pdx1*. Schematic representations of the cloned lentiviral plasmids are illustrated in Supplementary Figure 1b.

### 2.12. Lentiviral Vector Propagation and Titration

Lentiviral plasmids were cotransfected into HEK293T cells using calcium phosphate precipitation as previously described [3–5]. Lentiviral particles were harvested at 36, 48, 60, and 72 h posttransfection, filtered through Millex-HV 0.45 *μ*M polyvinylidene fluoride syringe filters (EMD Millipore^©^, Burlington, USA), and concentrated using Amicon Ultra 100 kDa filters (EMD Millipore^©^, USA). Concentrated lentiviral particles were titered using NIH3T3 cells and FACS analysis of eGFP and mCherry expression. Flow cytometry data were analyzed using BD FACSDiva™ software (Version 8.0.1).

### 2.13. Viral Transduction

Midpassage MSC-*Luc2* (1 × 10^5^ cells/well) were transduced overnight (MOI = 10) with HMD, HMD-*INS-FUR*, HMD-*Neurod1*, HMD-*Neurod1/INS-FUR,* and HMD-*Pdx1* in standard MSC medium supplemented with 8 *μ*g/ml Polybrene (Sigma-Aldrich™, Australia). Following transduction, lentiviral particles were removed and the cells cultured for 72 h, after which the cells were sorted into eGFP^+^ and mCherry^+^ populations at the Advanced Cytometry Facility (Centenary Institute, Sydney, Australia) using a BD FACSAria™ II and analysed using BD FACSDiva™ software (Version 6.1.3). Fluorescence imaging of positively transduced MSCs was performed using a Leica^©^ DM microscope (Leica Microsystems^©^, Australia).

### 2.14. Chronic and Acute Insulin Secretion

For chronic insulin secretion, untransduced and transduced MSCs (1 × 10^5^ cells/well in triplicate, *n* = 4) were cultured for 24 h in standard MSC medium. For glucose-stimulated insulin secretion, untransduced and transduced MSCs (1 × 10^5^ cells/well in triplicate, *n* = 3) were seeded in 6-well plates and sequentially stimulated with 20 mM D-Glucose (Sigma-Aldrich™, Australia), as previously described [25]. The human insulin concentrations in harvested supernatants, from both chronic and acute insulin secretion assays, were quantified using the ARCHITECT™ i4000SR Immunoassay Analyser (Abbott Diagnostics^®^, Macquarie Park, Australia). Data were represented as mean insulin concentration (pmol/ml/1 × 10^5^ cells) ± SD.

### 2.15. Induction of Diabetes in NOD/*Scid* Mice

NOD/*Scid* mice received 170 mg/kg of STZ in 0.1 M sodium citrate buffer (pH 4.0) via i.p. injection. All animals, including nondiabetic controls, had their body weights and blood glucose concentrations measured daily using an Accu-Chek^®^ Performa glucometer (Accu-Chek^®^, Roche, Castle Hill, Australia). Animals that did not develop hyperglycaemia (blood glucose concentration >8 mmol/l) within 1 week post-STZ injection received a second low-dose (40 mg/kg) STZ injection. Animals that displayed hyperglycaemia for four consecutive days were considered diabetic and were used for *in vivo* experiments.

### 2.16. Transplantation of MSC in STZ-NOD/*Scid* Mice

Two groups of STZ-diabetic NOD/*Scid* mice received s.c. injections of 1 × 10^7^ (*n* = 6) and 5 × 10^7^ (*n* = 6) late passage *INS-FUR*-expressing MSCs, respectively. Nondiabetic (*n* = 6) and untreated diabetic (*n* = 6) animals were assessed alongside treated animals. Body weights and blood glucose concentrations were measured daily. Animals that displayed hypoglycaemia (blood glucose concentration <3 mmol/l) or body weight loss (>10%) for two consecutive days were euthanized by CO_2_ asphyxiation and cervical dislocation.

### 2.17. Intraperitoneal Glucose Tolerance Test (IPGTT)

Normal (*n* = 5) and treated (*n* = 3) mice fasted for 6 h, were transferred to a ZDS Qube Manifold 5 Station (Advanced Anesthesia Specialists^®^, Australia), and were maintained under stable anaesthesia (2.5 l/min isoflurane and 1.5 l/min O_2_). Mice received an i.p. injection of 2 g/kg 50% v/v liquid glucose (0.5 g/ml). Blood glucose was measured at 0, 5, 15, 30, 60, and 90 min postinjection. Following IPGTTs, animals were euthanized by CO_2_ asphyxiation and cervical dislocation.

### 2.18. Statistical Analysis

All statistical analysis was performed using GraphPad Prism 7^®^ software. Values were presented as means ± SDs or SEMs. One-way or two-way ANOVA, with the appropriate posttests, was performed, with *p* < 0.05 indicating significance.

## 3. Results

### 3.1. *In Vitro* Characteristics of NOD MSCs

**Figure 1:**
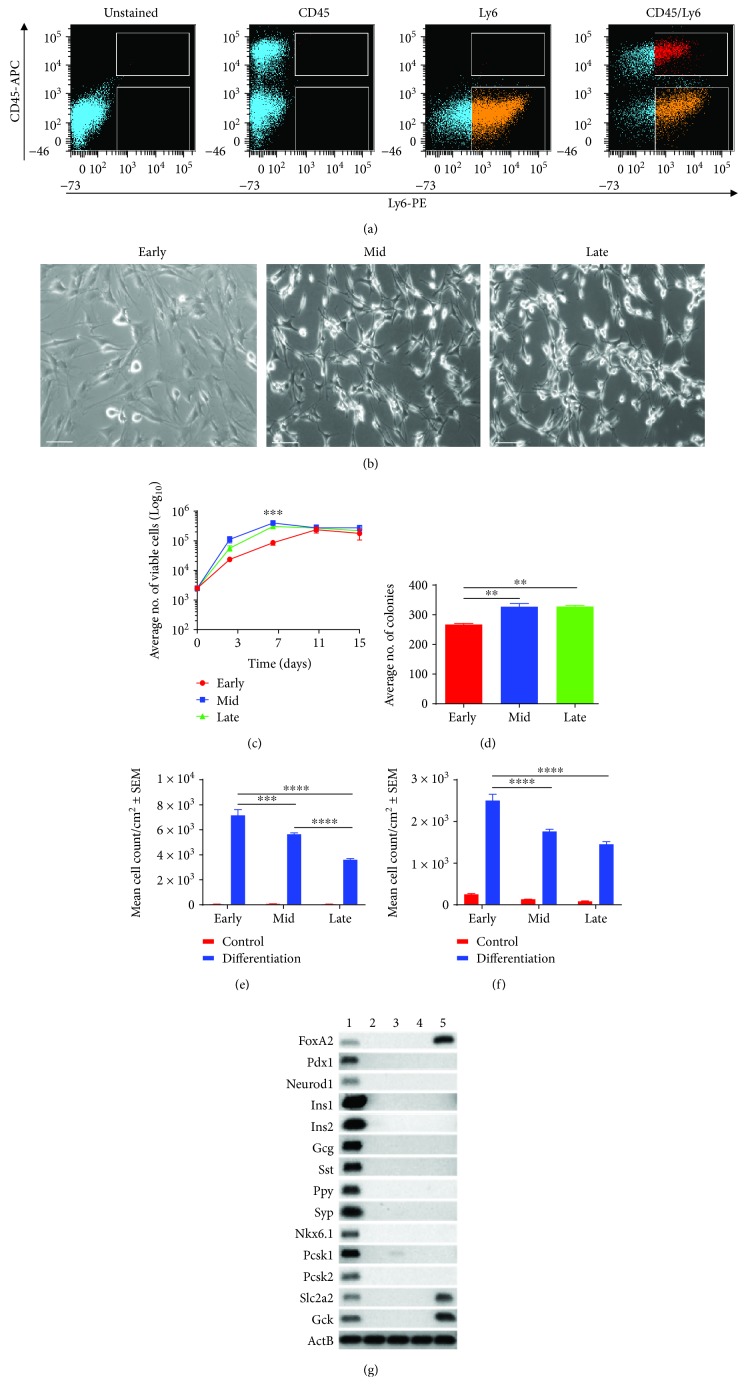
*In vitro* characteristics of NOD-derived MSCs with cell culture expansion. (a) FACS analysis and enrichment of NOD-derived MSCs. Following culture for two passages, NOD bone marrow stromal cells were stained with nil antibody (unstained), CD45 mAb conjugated to fluorochrome APC (CD45-APC), Ly6 MAb conjugated to fluorochrome PE (Ly6-PE), and both mAbs (CD45-APC/Ly6-PE). Fluorescence dot plots of CD45-APC (*y*-axis) and Ly6-PE (*x*-axis) were used to identify the MSC (CD45^−^/Ly6^+^; orange) and double-positive (CD45^+^/Ly6^+^; red) cell subpopulations ready for cell sorting using the BD FACSAria™ II instrument. Representative of three individual FACS experiments. (b) Plastic adherence, fibroblast-like morphology, and self-renewal without differentiation into other cell types. MSCs maintained fibroblast-like morphology as assessed using light microscopy (Leica DM microscope; 10x magnification; scale bar = 100 *μ*M). (c) Improved cell proliferation with culture expansion. Data are presented as mean viable cells ± SDs (*n* = 3). A two-way ANOVA with Tukey's posttests was performed, ^∗^ *p* < 0.05. (d) Improved fibroblastic colony formation following methylene blue staining. Data are presented as mean number of colonies ± SEMs (*n* = 3). A one-way ANOVA and Tukey's posttests were performed, ^∗^ *p* < 0.05. (e) Semiquantitative analysis of adipogenic differentiation under defined conditions. NOD-derived MSCs maintained fat formation following Oil Red O staining, albeit at reduced levels, with increasing passage number. Data are presented as mean cell count/cm^2^ ± SEM (*n* = 3). A two-way ANOVA and Tukey's posttests were performed, ^∗^ *p* < 0.05. (f) Semiquantitative analysis of osteogenic differentiation under defined conditions. NOD-derived MSC maintained bone formation following Alizarin Red staining, albeit at lower levels, with increasing passage number. Data are presented as mean cell count/cm^2^ SEM (*n* = 3). A two-way ANOVA and Tukey's posttests were performed, ^∗^ *p* < 0.05. (g) Pancreatic transcription factor and hormone and protein expression levels were determined by RT-PCR. MSCs did not express any transcription factors, hormones, or protein found in the pancreas. Positive control mouse pancreas (lane 1), plastic-adherent MSCs (lane 2), plastic-adherent hematopoietic cells (lane 3), adherent bone marrow cells (lane 4), and negative control mouse liver (lane 5).

MSCs identified by FACS were characterized by the surface marker profile CD45^−^/Ly6^+^ and constituted ~33% of the parental stromal cell population (Figure 1(a)). MSCs displayed the characteristic fibroblast-like morphology from early to late passage numbers (Figure 1(b)). However, a decrease in MSC diameter was observed with increasing passage, from ~100 *μ*m (early passage) to 50 *μ*m (late passage). Although MSCs underwent a period of early passage replicative crisis during P5-8 (data not shown), MSCs continued to self-renew up to 60 passages (maximum culture period). An intrapopulation analysis of MSC proliferation and clonogenicity showed no significant difference in proliferation (Figure 1(c)) and conservation of clonogenicity potency (Figure 1(d)) from early to late passage numbers.

To demonstrate that NOD MSCs underwent trilineage differentiation, as defined by the International Society Cell and Gene Therapy (ISCT), trilineage differentiation assays were performed at early, mid, and late passage numbers. NOD MSC demonstrated trilineage differentiation into adipocytes (Supplementary Figure 2), osteocytes (Supplementary Figure 3), and chondrocytes (Supplementary Figure 4). Semiquantitative analysis of adipogenesis and osteogenesis was assessed by scoring the degree of differentiation, as previously described [24]. MSCs displayed reduced adipogenesis (Figure 1(e)) and osteogenesis (Figure 1(f)) with increasing passage number.

To confirm that MSCs did not intrinsically express any pancreatic transcription factors or hormones, RT-PCR analyses were performed and showed a lack of expression of pancreatic transcription factors, hormones, and proteins in all adherent bone marrow cell populations (Figure 1(g)). As expected, all genes were expressed in normal mouse pancreas (positive control), and *FoxA2*, *Scl2a2*, and *Gck* were expressed in normal mouse liver (negative control).

### 3.2. Syngeneic MSCs Are Cleared in an Immune-Competent Animal Model

**Figure 2:**
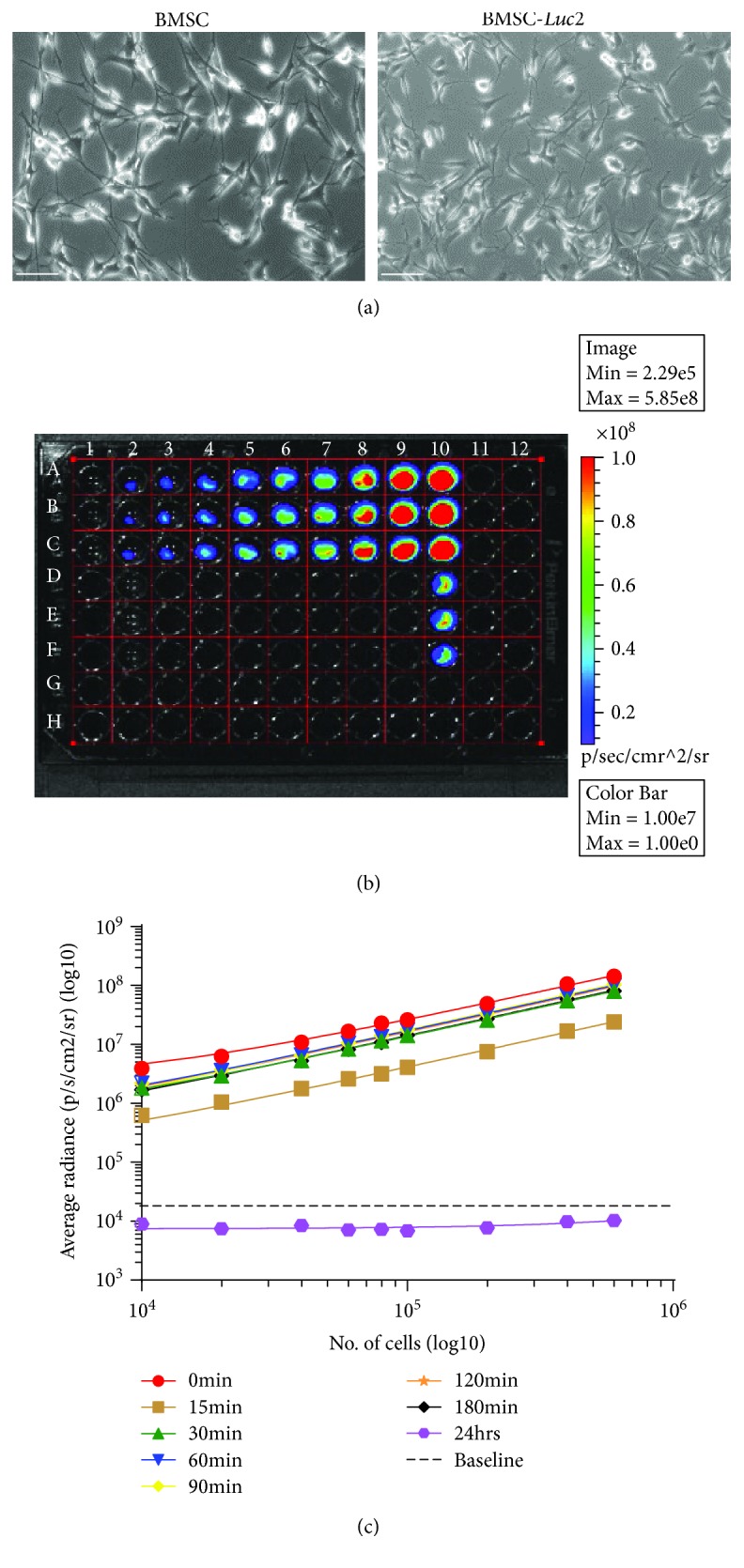
NOD-derived MSC nucleofection. (a) MSCs (early passage number) were nucleofected with 0 and 5 *μ*g pVITRO2-*Luc2*. Parental MSCs and MSC-*Luc2* at an equivalent passage number (P15) showed native fibroblast-like morphology and maintained plastic adherence and self-renewal properties. Images were acquired on a Leica DM light microscope at 10x magnification, scale bar = 100 *μ*m. (b) *In vitro* functional characterization of luciferase activity in MSC-*Luc2.* Cells were incubated with 1 : 1 D-luciferin (300 *μ*g/ml) and imaged on the IVIS Lumina II, according to the *in vitro* BLI acquisition settings. The image represented is at *t* = 30 min after the addition of D-luciferin. Lane 1: D-PBS; lanes 2-10: MSC-*Luc2* and MSC-*Luc2/LacZ* (control). (c) Linear regression analysis of luminescent signal was performed using GraphPad Prism 7^®^. Data are presented as means ± SDs of triplicates.

Noninvasive BLI is an established and sensitive tool for assessing cell replacement therapy safety and efficacy in living preclinical small animal models. Furthermore, preclinical BLI results often serve as the decision point of the suitability of a cell replacement therapy for clinical trial testing in humans. This study utilized the Firefly luciferase reporter gene, *Luc2*, a *Luc2*-specific light-producing substrate D-luciferin, and an IVIS Lumina II imaging system (Perkin Elmer^©^). Prior to performing *in vitro* and *in vivo* BLI, a clonal population of MSCs expressing *Luc2* (MSC-*Luc2*) was obtained by selection with Hygromycin B. MSC-*Luc2* retained a fibroblast-like morphology similar to that observed for parental MSCs (Figure 2(a)); however, these cells exhibited a reduced cell diameter (~100 *μ*m nucleofected versus 150 *μ*m parental). *In vitro* analyses of BLI at multiple time points over a 3-hour period (Figure 2(b)), in combination with linear regression analysis (Figure 2(c)), confirmed that a clonal population of MSC-*Luc2* had been selected and that bioluminescence was stable for up to 3 h *in vitro*.

**Figure 3:**
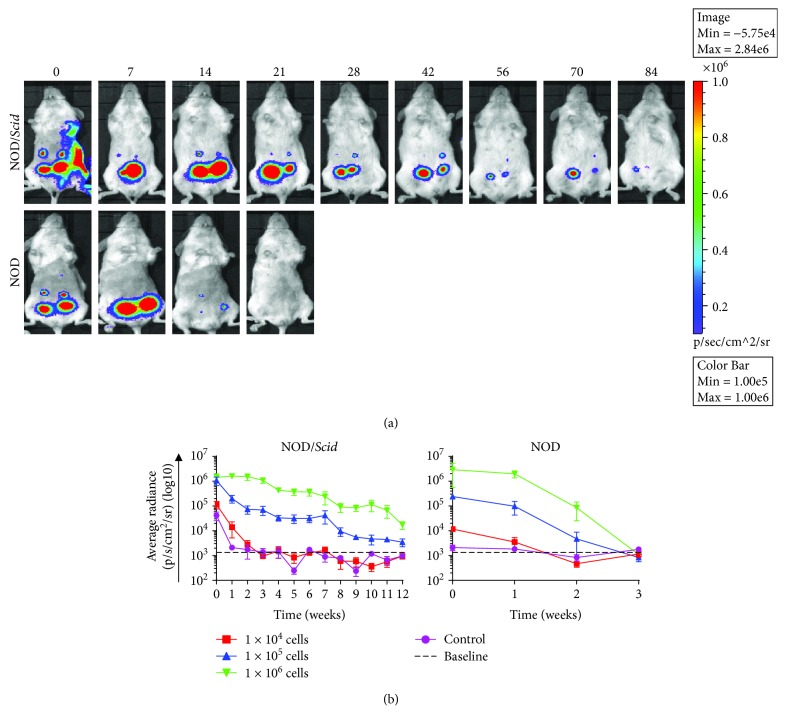
Persistence of syngeneic MSCs in immune-competent and immune-deficient animal models. (a) NOD (*n* = 4) and NOD/*Scid* (*n* = 4) mice (6-10 weeks of age) received a total of six subcutaneous (s.c.) injections of 1 × 10^4^ (*n* = 2), 1 × 10^5^ (*n* = 2), and 1 × 10^6^ (*n* = 2) midpassage MSC-*Luc2* cells/mouse. Untreated age-matched NOD (*n* = 2) and NOD/*Scid* (*n* = 2) mice were utilized as negative controls. BLI images were acquired following i.p. administration of D-luciferin (15 mg/ml) at 150 mg/kg or 10 *μ*l/g. Images are representative of a single experimental NOD/*Scid* and NOD animal. (b) Analysis of MSC BLI in NOD and NOD/*Scid* mice. Regions of interest were established surrounding the areas corresponding to the sites of cell transplantation using the Living Image 3.1 (PerkinElmer™) software. Quantitative data were subsequently analyzed using GraphPad Prism 7^®^. Data were presented as mean radiance ± SEM over time (weeks). A two-way ANOVA and Tukey's posttests were performed, ^∗^ *p* < 0.05.

We chose to assess the persistence of MSCs transplanted subcutaneously in immune-competent NOD and immune-deficient NOD/*Scid* animal models as this most closely reflects the route of administration of a cell replacement therapy for individuals with T1D. Thus, MSC-*Luc2* were transplanted subcutaneously at multiple cell concentrations to determine the lowest cell concentration and the length of time for which bioluminescence could be detected (Figure 3(a)). Quantitative analysis of BLI data showed that in both NOD/*Scid* and NOD mice there was an increase in bioluminescence with increasing cell dose (Figure 3(b)), which resulted in a dose-dependent increase in persistence of bioluminescence in both animal models (Supplementary Tables 2A and 2B). Bioluminescence, albeit diminished, could be detected in NOD/*Scid* mice for up to 12 weeks, suggesting poor survival of MSCs at the s.c. transplant site. By comparison, bioluminescence persisted for 2 weeks in NOD mice, after which signal could no longer be detected, suggesting an immune-mediated clearing of the MSC graft. In fact, upon challenge with a follow-up injection of 1 × 10^6^ MSCs, clearing of the MSC graft occurred within 1 week postinjection (data not shown). These kinetics are consistent with the generation of memory T cell populations stimulated after the initial exposure to MSCs.

### 3.3. *Neurod1* and *Pdx1* Fail to Induce *β*-Cell Differentiation of *Ex Vivo* Expanded NOD MSCs

**Figure 4:**
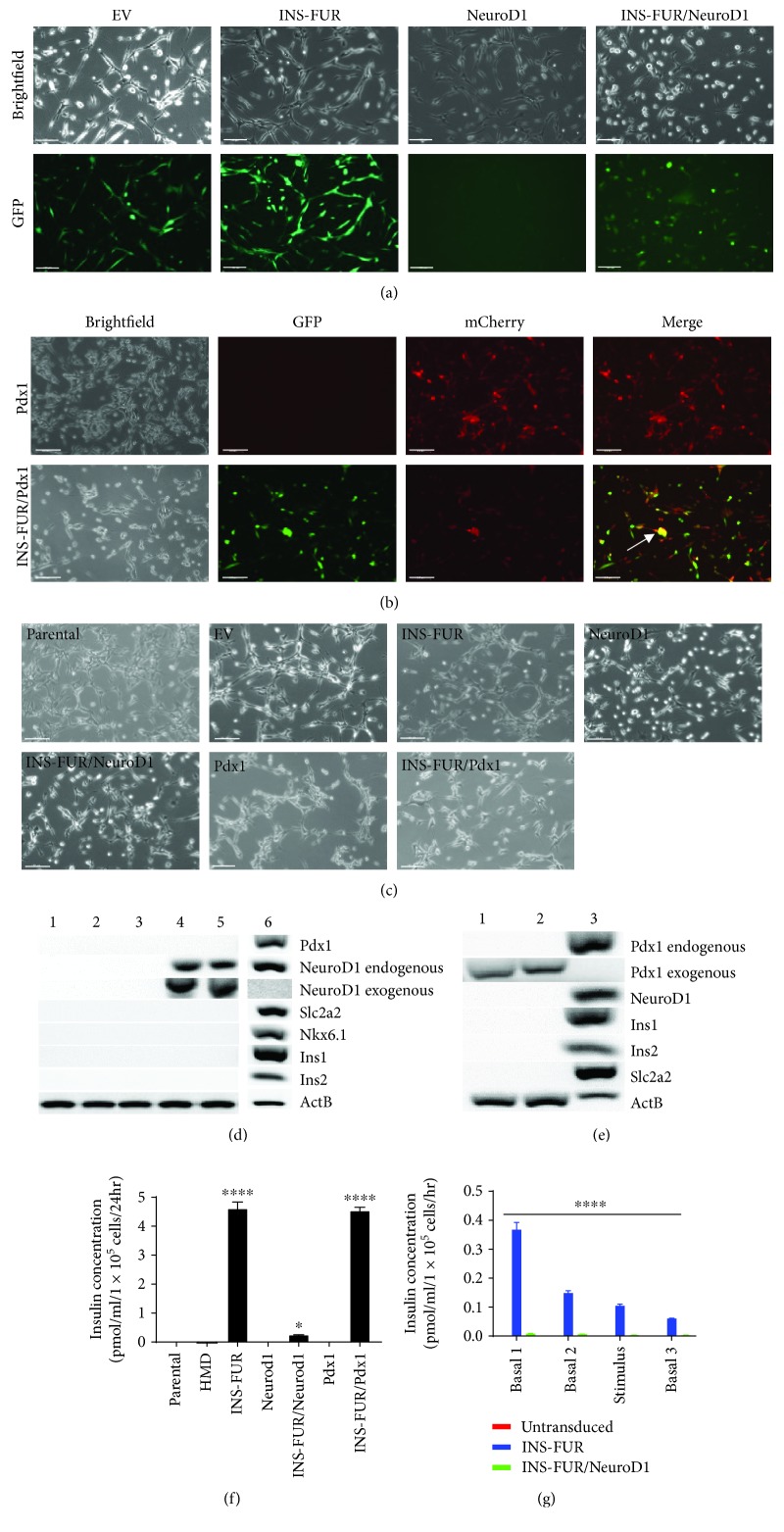
Analysis of *INS-FUR-*, *Neurod1-*, and *Pdx1*-transduced MSC. (a) Fluorescence imaging of MSCs transduced with empty vector (EV), *Neurod1*, and *INS-FUR*. Transduced cells were returned to culture after sorting for GFP positivity and imaged for GFP expression (day 7 posttransduction) using a Leica DM fluorescence microscope (Leica Microsystems), 10x magnification with bright field and GFP fluorescence filter sets; scale bar = 100 *μ*m. (b) Fluorescence imaging of MSCs and MSC-*INS-FUR* transduced with *Pdx1*. Transduced cells were returned to culture post sorting and imaged for GFP and mCherry expression (day 7 posttransduction) using a Leica DM microscope, 10x magnification under bright field, GFP and Texas Red fluorescence filter sets; scale bar = 100 *μ*m. (c) Morphological characterization of MSCs posttransduction. Bright-field images were acquired at 28 days posttransduction on a Leica DM microscope (Leica Microsystems) at 10x magnification under bright-field setting; scale bar = 100 *μ*m. (d) Gene expression profiling of *INS-FUR* and *Neurod1*-expressing MSC. Lane 1: untransduced MSC; lane 2: MSC-*EV*; lane 3: MSC-*INS-FUR*; lane 4: MSC-*Neurod1*; lane 5: MSC-*INS-FUR/Neurod1*, and lane 6: mouse pancreas (positive control). (e) Gene expression profiling of *Pdx1*-expressing MSCs. Lane 1: MSC-*Pdx1*; lane 2: MSC-*INS-FUR/Pdx1*; and lane 3: mouse pancreas (positive control). (f) Chronic insulin secretion from transduced MSCs. Human insulin was quantified using the ARCHITECT™ i4000SR Immunoassay Analyser (Abbott Diagnostics^©^). Data are represented as means ± SDs (*n* = 4). A one-way ANOVA with Sidak's posttests were performed, ^∗^ *p* < 0.05. (g) Acute glucose-stimulated insulin secretion from *INS-FUR*- and *Neurod1*-transduced MSCs. Human insulin was quantified using the ARCHITECT™ i4000SR Immunoassay Analyser (Abbott Diagnostics^©^). Data were presented as means ± SDs (*n* = 3). Two-way ANOVA with Tukey's posttests were performed, ^∗^ *p* < 0.05.

The pancreatic transcription factors, *Neurod1* and *Pdx1*, were overexpressed in MSC-*Luc2* to function as mediators of pancreatic transdifferentiation, whilst the *INS-FUR* gene was overexpressed to allow for mature human insulin production. Transduced MSCs were analysed via FACS and sorted into individual populations, as outlined in Supplementary Tables 3A and 3B. The sorted MSCs were returned to culture and imaged for eGFP and mCherry expression 7 days posttransduction (Figures 4(a) and 4(b)). As can be seen, eGFP expression in MSCs transduced with *Neurod1* and *INS-FUR/Neurod1* was lower than that in cells transduced with the existing HMD and HMD-*INS-FUR* lentiviral vectors, a consequence of lower viral titers.

Transduced MSCs were subsequently cultured for a period of 28 days, at which point morphological analysis of the differentiation process was performed (Figure 4(c)). Transduced MSCs expressing both murine *Neurod1* and *Pdx1* alone, or in combination with *INS-FUR*, retained a fibroblast-like morphology in comparison to their untransduced counterparts, demonstrating that no change in morphology was attributable to transgene overexpression.

Gene expression profiling was performed to determine if ectopic expression of *Neurod1*, *Pdx1*, and *INS-FUR* in *ex vivo* expanded MSCs resulted in pancreatic differentiation. As can be seen in Figure 4(d), exogenous murine *Neurod1* was detected at 28 days posttransduction in MSCs transduced with HMD-*Neurod1* and HMD-*INS-FUR/Neurod1*, and as expected, expression was not detected in the parental MSCs, MSCs transduced with HMD and HMD-*INS-FUR*, and the positive control (mouse pancreas). Interestingly, exogenous *Neurod1* expression resulted in the expression of endogenous *Neurod1*, suggesting a potential autoregulatory function of *Neurod1*. However, *Pdx1*, *Nkx6.1*, *Scl2a2*, *Ins1*, and *Ins2* were not detected in parental and transduced MSCs. In Figure 4(e), a similar pattern of gene expression was observed following the ectopic expression of murine *Pdx1*, as was observed with ectopic expression of *Neurod1*, with the exception that ectopic *Pdx1* did not result in endogenous *Pdx1* expression. Together, these data confirm the lack of pancreatic differentiation in *ex vivo* expanded MSC expressing *Neurod1* and *Pdx1*.

### 3.4. Gene-Modified *Ex Vivo* Expanded NOD MSCs Demonstrate Abnormal Glucose-Stimulated Insulin Secretion

The ability of transduced MSCs to secrete mature insulin *in vitro* in response to glucose stimulation was determined. A significant quantity of mature insulin (~4.5 pmol/ml/1 × 10^5^ cells) was detected in the medium of MSCs expressing *INS-FUR* alone and in combination with *Pdx1*. By comparison, untransduced MSCs and MSCs transduced with empty vector or *Neurod1* alone did not secrete mature insulin (Figure 4(f)). There was also a significant difference (*p* < 0.005) in the quantity of insulin secreted between MSC*-INS-FUR* (~4.5 pmol/ml/1 × 10^5^ cells) and MSC*-INS-FUR/Neurod1* (~0.3 pmol/ml/1 × 10^5^ cells), which directly correlated with differences in the expression of the fluorescent reporter. Acute glucose stimulation assays showed that following stimulation with 20 mM D-Glucose, glucose-stimulated insulin secretion (GSIS) was not present (Figure 4(g)). The absence of GSIS was expected due to the lack of *Slc2a2* expression, as detected by RT-PCR.

### 3.5. *INS-FUR*-Expressing MSCs Fail to Restore Normoglycaemia in STZ-Diabetic NOD/*Scid* Mice

**Figure 5:**
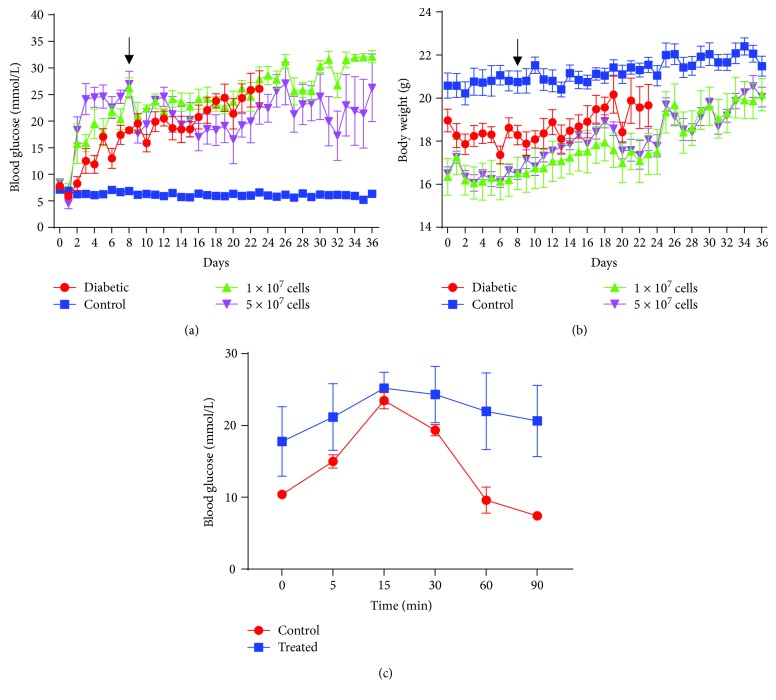
Transplantation of MSC-*INS-FUR* in STZ-diabetic NOD/*Scid* mice. (a) Blood glucose concentrations of treated STZ-diabetic NOD/*Scid* mice. Blood glucose measurements were recorded daily post-STZ injection for the duration of the experiment (36 days). Data were presented as means ± SEMs (*n* = 6 mice per group). Two-way ANOVA with Sidak's posttests were performed, ^∗^ *p* < 0.05. (b) Body weights of treated STZ-NOD/*Scid* mice. Body weight measurements were recorded daily post-STZ injection for the duration of the experiment (36 days). Data were presented as means ± SEMs (*n* = 6 mice per group). Two-way ANOVA was performed with Sidak's posttests, ^∗^ *p* < 0.05. (c) IPGTT in treated STZ-diabetic NOD/*Scid* mice. Blood glucose measurements were obtained at 0, 5, 15, 30, 60, and 90 min post-D-Glucose injection. Data were presented as means ± SEMs (*n* = 5, normal; *n* = 3, treated). Two-way ANOVA with Sidak's posttests were performed, ^∗^ *p* < 0.05.

To determine if *INS-FUR*-expressing MSCs could restore normoglycaemia in STZ-diabetic NOD/*Scid* mice, animals received a s.c. transplant of either 1 × 10^7^ or 5 × 10^7^ cells. In animals treated with 5 × 10^7^ cells, within 24 h following transplantation, there was a decrease in blood glucose concentrations, and a significant (*p* < 0.05) decrease was observed at days 8 and 12 posttransplantation (Figure 5(a)). In addition, there was a significant decrease in the blood glucose levels of the animals treated with 5 × 10^7^ cells, from before to after transplantation, for a period of ~15 days (pretransplant vs. posttransplant, *p* < 0.05) (Supplementary Tables 4A and 4B). By comparison, blood glucose concentrations in diabetic animals and animals treated with 1 × 10^7^ cells remained significantly higher (*p* < 0.0001) than those in the normal controls for the duration of the experiment. Most importantly, animals treated with either 1 × 10^7^ or 5 × 10^7^ cells did not normalize blood glucose concentration at any time for the duration of the experiment. In addition, there was a significant difference in the body weights of treated animals in comparison to normal controls both before and after transplantation, despite random allocation of animals to the different groups (Figure 5(b)). However, at no time point was there a significant decrease in body weight observed over the time course of the experiment.

Prior to euthanasia, animals treated with 5 × 10^7^ cells (*n* = 3) were assessed for glucose tolerance via an IPGTT. Treated animals displayed an abnormal glucose tolerance in comparison to normal controls (Figure 5(c)). The high degree of variation in the glucose tolerance observed for the treatment group was due to one treated animal beginning the IPGTT at a significantly lower blood glucose concentration than the other treated animals, a consequence of the cell transplant having successfully reduced blood glucose levels in this animal.

## 4. Discussion

Stem cells are characterized by two defining long-term characteristics: (i) the ability for renewal without differentiation into other cell types when cultured under standard conditions and (ii) the continued potential to develop into specialized cell types when cultured under defined experimental conditions. The early, mid, and late passage number NOD-derived MSCs utilized in this study fulfilled the ISCT criteria of plastic adherence, self-renewal, and colony formation without differentiation into other cell types under standard MSC culture conditions and trilineage mesenchymal differentiation into bone, fat, and cartilage under defined cell culture conditions [20, 21].

In this study, NOD-derived MSCs maintained a fibroblast-like morphology, potent self-renewal, and clonogenicity throughout early, mid, and late passage numbers. This finding is in contrast to previous studies, which suggest that MSCs undergo age-related changes with continued cell passage [26–28]. The maintenance of a fibroblast-like morphology can be attributed, in part, to medium supplementation with bFGF. These results are supported by another study, where bFGF inhibited apoptosis and promoted proliferation of MSCs through a reduction in cellular oxidative stress [29]. Although MSC characteristics of self-renewal and colony-forming units (CFU-F) were conserved following cell culture expansion, MSCs showed some reduction in trilineage differentiation with increasing passage, which is in concordance with other studies [30–32]. Furthermore, several studies have demonstrated the detrimental effect of aging on multipotential differentiation, proliferation, and senescence [31, 33, 34]. Collectively, these findings highlight the importance of ongoing surveillance of stem cell-like properties, amongst other defining characteristics, when engineering replacement cell therapies.

To clarify whether NOD-derived MSCs were immune-evasive, we subcutaneously transplanted syngeneic MSCs into immune-competent NOD mice, which were detectable by BLI up to 14 days posttransplantation. In a similar study, MSCs that were transduced to coexpress luciferase and GFP, subsequently transplanted via the kidney artery in BALB/c mice, were detected via BLI up to 14 days posttransplantation [35]. This demonstrates that the site of cell administration in immune-competent models does not affect cell survival and that syngeneic MSCs do not appear to be immune-privileged [36]. This may be due to several factors, including the overexpression of the nonmammalian *Luc2* transgene and contaminating FBS, factors that may override the inherent immune privilege characteristics of MSCs. In fact, previous studies have demonstrated that sustained high levels of luciferase expression induce luciferase-specific immune responses in immune-competent animal models, thereby limiting the utility of luciferase as an *in vivo* reporter in transplantation studies [37, 38].

However, in immune-deficient NOD/*Scid* mice, syngeneic MSCs were detected for a significantly longer period (up to 12 weeks posttransplantation), albeit with diminishing persistence. These results are supported by similar studies, which show loss of transplanted MSCs in immune-deficient animal models [39–41]. The current study highlights the importance of reporter gene selection and the limited timeframe in which MSC therapeutic effects can be evaluated, in both immune-competent and immune-deficient animal models. Assessment of MSCs in short-term studies has demonstrated their protective benefits [42], whereas long-term studies show little or no protection [43], which may be attributable to their lack of persistence in immune-competent models.

We also assessed the ability to differentiate *ex vivo* expanded MSCs into surrogate *β*-cells. Considering the success of *Neurod1* as a mediator of *β*-cell differentiation in nonpancreatic tissues, we sought to determine the potential of *Neurod1* to induce *β*-cell differentiation of MSCs. In transduced MSCs, overexpression of *Neurod1* and *INS-FUR* did not result in the cuboidal morphological changes associated with *β*-cell differentiation. In addition, gene expression profiling confirmed a lack of *β*-cell differentiation due to the absence of expression of endogenous pancreatic transcription factors and insulin. A recent study by Qing-Song et al. assessed the effect of *Pdx1*, *Neurod1*, and *MafA* overexpression in MSCs and showed that *Neurod1* was a weak inducer of endogenous *Pdx1* and *Ins2* expression and that only in combination with *Pdx1* and *MafA* was there a significant induction of *β*-cell differentiation and insulin expression [44]. Surprisingly, in this study, the lack of *β*-cell differentiation following overexpression of *Pdx1* in culture-expanded MSCs indicated that *ex vivo* expansion results in defects in the pancreatic differentiation potential of MSCs.

A previous study showed that *in vivo* transplantation is required for functional *β*-cell maturation [45]; therefore, *INS-FUR*-expressing MSCs were assessed for their ability to reverse diabetes following transplantation into STZ-diabetic NOD/*Scid* mice. Upon transplantation of 5 × 10^7^ *INS-FUR*-expressing MSCs, blood glucose concentrations decreased from 25-30 to 15-20 mmol/l. However, at no time point did blood glucose levels fall within the normal physiological range (5-7 mmol/l). This is likely due to the severe hyperglycaemia induced in these animals, which likely requires higher cell numbers to restore blood glucose levels to within the normal physiological range. Despite this, there was a significant decrease in the blood glucose levels of treated vs. diabetic animals (*p* < 0.05) that was maintained for ~2 weeks; after which, blood glucose concentrations began to increase to pretransplant values. The subsequent increase in blood glucose concentrations of treated animals correlated with the results of the MSC persistence studies in NOD/*Scid* mice. In addition, IPGTTs of transplanted animals showed that they exhibited abnormal glucose tolerance, indicating a lack of *in vivo* GSIS. This finding corroborated the *in vitro* characterization studies.

In conclusion, the results of this study highlight several caveats to MSC-based gene therapy for T1D, which warrant careful consideration prior to the formulation of clinical trials. Considering that overexpression of *Pdx1* in early-passage MSCs results in pancreatic differentiation [14], these data show that *ex vivo* expansion impairs pancreatic differentiation of NOD-derived MSCs through the age-related loss of multipotency. Therefore, gene modification should be performed as soon as practicable after the isolation of MSCs [46]. In addition, given that *ex vivo* expansion is required to generate sufficient quantities of adult-derived MSCs for therapeutic purposes and that this process impairs their therapeutic potential, the use of embryonic stem cells or induced pluripotent stem cells as an unlimited stem cell source may overcome this limitation [47, 48].

## Data Availability

The data used to support the findings of this study are available from the corresponding author upon request.

## Supplementary Materials

Supplementary Materials Supplementary Figure 1: schematic of mammalian and lentiviral expression plasmid. (a) The luciferase gene was excised from pGL4.20 (Luc2/Puro) plasmid and subcloned into MCS1 of pVITRO2-hygro-mcs to generate the bioluminescent plasmid pVITRO2-*Luc2*. (b) The lentiviral plasmids HMD and HMD-*INS-FUR* were existing plasmids from a previous study. The *Neurod1-T2A-eGFP* sequence was subcloned to replace the existing eGFP sequence in the HMD and HMD-*INS-FUR* plasmids to generate HMD-*Neurod1* and HMD-*INS-FUR/Neurod1*, respectively. The *Pdx1-IRES-mCherry* sequence was subcloned to replace the existing eGFP sequence of the HMD vector to generate HMD-*Pdx1*. Supplementary Figure 2: *in vitro* NOD-derived MSC adipogenesis, osteogenesis, and chondrogenesis (a) NOD-derived MSCs at early, mid, and late passage numbers were cultured *in vitro* with control or adipogenic differentiation media. Lipid development in mature (white arrow) and immature adipocytes (yellow arrow) was assessed following Oil Red O staining. (b) NOD-derived MSCs at early, mid, and late passage numbers were cultured *in vitro* with control or osteogenic differentiation media. Calcium development (white arrows) was assessed with Alizarin Red staining. (c) NOD-derived MSCs at early, mid, and late passage numbers were cultured *in vitro* with control or chondrogenic differentiation media for 18 days. Filamentous glycosaminoglycan development during chondrogenesis (indicated by white arrows) was assessed with Alcian blue staining. Images were acquired on a Leica DM microscope, 20x magnification, scale bar = 100 *μ*m. Supplementary Table 1: list of oligonucleotide primers for RT-PCR. Supplementary Table 2A: average stained cell acquisition and analysis of NOD BMSCs and double-positive cells. Supplementary Table 2B: average purity analysis of sorted BMSCs and double-positive cells. Supplementary Table 3A: persistence of bioluminescent signal in NOD/*Scid* mice. Supplementary Table 3B: persistence of bioluminescent signal in NOD mice. Supplementary Table 4A: cell acquisition analysis of lentivirus-transduced BMSCs. Supplementary Table 4B: sorted cell purity analysis of lentivirus-transduced BMSCs. Supplementary Table 5A: significance in blood glucose concentration following BMSC-*INS-FUR* transplant compared to normal controls. Supplementary Table 5B: significance in body weight following BMSC-*INS-FUR* transplant compared to normal controls.
