# Supplementary material for: Ex Vivo Expansion of Murine MSC Impairs Transcription Factor-Induced Differentiation into Pancreatic β-Cells
Source: Stem Cells Int. 2019 Mar 10;2019:1395301. doi: 10.1155/2019/1395301 (PMC6431458; doi:10.1155/2019/1395301)
Supplement: Supplementary Materials — Supplementary Figure 1: schematic of mammalian and lentiviral expression plasmid. (a) The luciferase gene was excised from pGL4.20 (Luc2/Puro) plasmid and subcloned into MCS1 of pVITRO2-hygro-mcs to generate the bioluminescent plasmid pVITRO2-Luc2. (b) The lentiviral plasmids HMD and HMD-INS-FUR were existing plasmids from a previous study. The Neurod1-T2A-eGFP sequence was subcloned to replace the existing eGFP sequence in the HMD and HMD-INS-FUR plasmids to generate HMD-Neurod1 and HMD-INS-FUR/Neurod1, respectively. The Pdx1-IRES-mCherry sequence was subcloned to replace the existing eGFP sequence of the HMD vector to generate HMD-Pdx1. Supplementary Figure 2: in vitro NOD-derived MSC adipogenesis, osteogenesis, and chondrogenesis (a) NOD-derived MSCs at early, mid, and late passage numbers were cultured in vitro with control or adipogenic differentiation media. Lipid development in mature (white arrow) and immature adipocytes (yellow arrow) was assessed following Oil Red O staining. (b) NOD-derived MSCs at early, mid, and late passage numbers were cultured in vitro with control or osteogenic differentiation media. Calcium development (white arrows) was assessed with Alizarin Red staining. (c) NOD-derived MSCs at early, mid, and late passage numbers were cultured in vitro with control or chondrogenic differentiation media for 18 days. Filamentous glycosaminoglycan development during chondrogenesis (indicated by white arrows) was assessed with Alcian blue staining. Images were acquired on a Leica DM microscope, 20x magnification, scale bar = 100 μm. Supplementary Table 1: list of oligonucleotide primers for RT-PCR. Supplementary Table 2A: average stained cell acquisition and analysis of NOD BMSCs and double-positive cells. Supplementary Table 2B: average purity analysis of sorted BMSCs and double-positive cells. Supplementary Table 3A: persistence of bioluminescent signal in NOD/Scid mice. Supplementary Table 3B: persistence of bioluminescent signal in NOD mice. Su [file 1395301.f1.pdf]

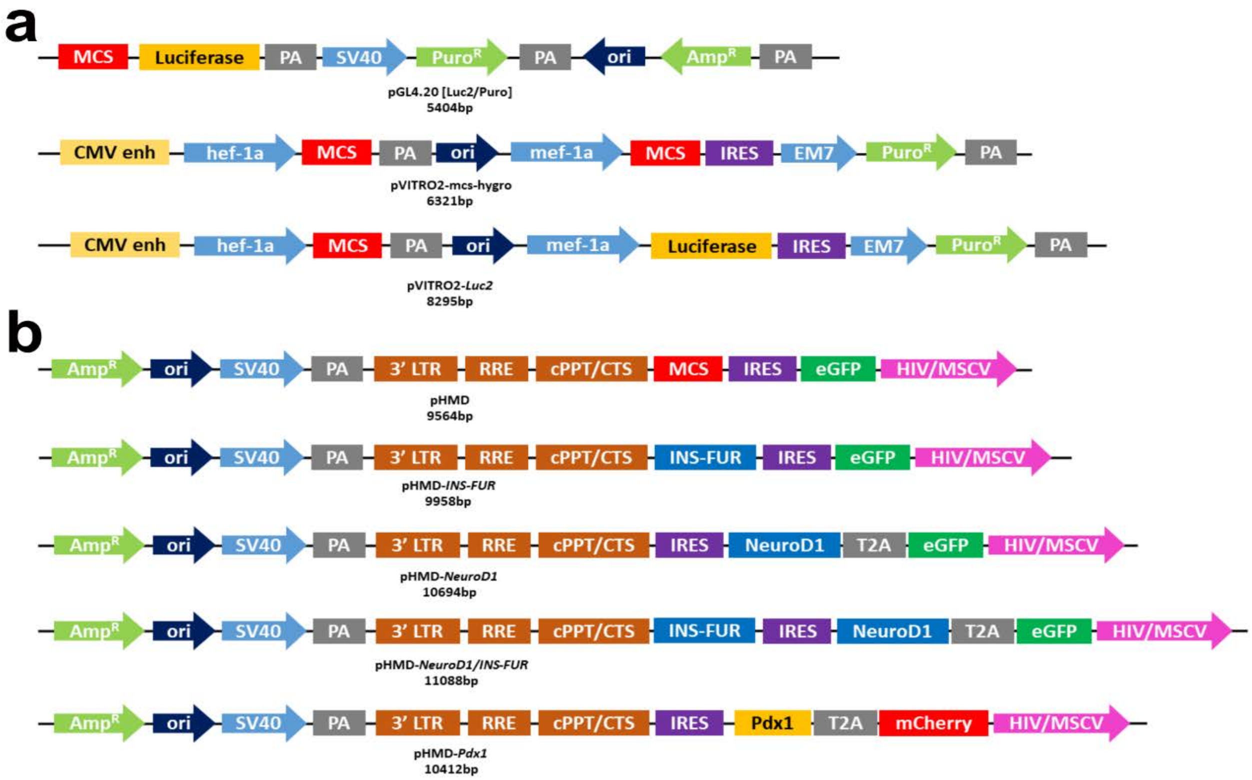

Supplementary Figure 1: Schematic of mammalian and lentiviral expression plasmid (a) The luciferase gene was excised from pGL4.20 [Luc2/Puro] plasmid and sub-cloned into MCS1 of pVITRO2-hygro-mcs to generate the bioluminescent plasmid pVITRO2-*Luc2*. (b) The lentiviral plasmids HMD and HMD-*INS-FUR* were existing plasmids from a previous study. The *Neurod1-T2A-eGFP* sequence was sub-cloned to replace the existing eGFP sequence in the HMD and HMD-*INS-FUR* plasmids to generate HMD-*Neurod1* and HMD-*INS-FUR/Neurod1,* respectively. The *Pdx1-IRES-mCherry* sequence was sub-cloned to replace the existing eGFP sequence of the HMD vector to generate HMD-*Pdx1*.

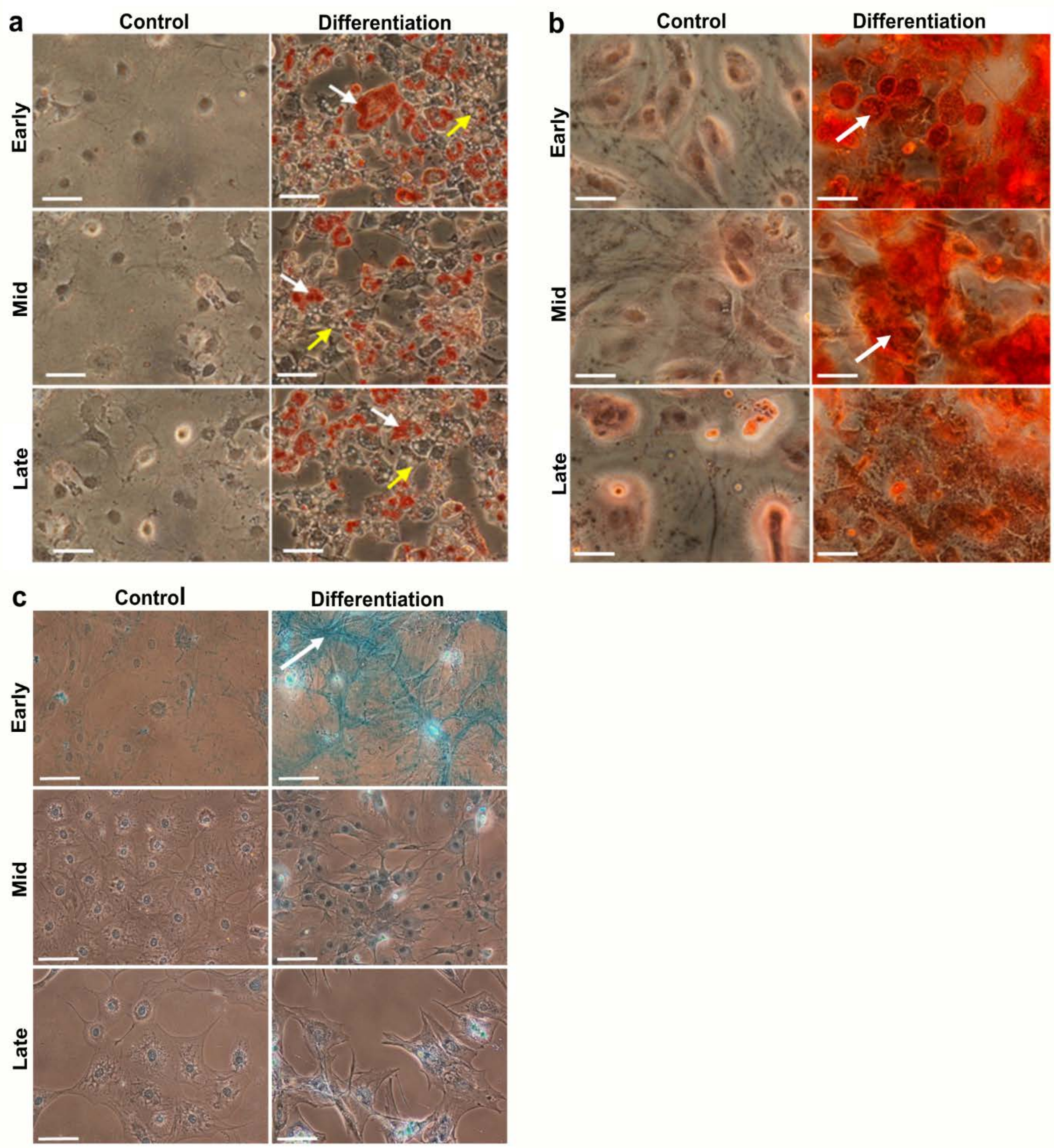

Supplementary Figure 2: ***In vitro*** **NOD derived MSC adipogenesis, osteogenesis and chondrogenesis** (s) NOD derived MSCs at early, mid and late passage number were cultured *in vitro* with control or adipogenic differentiation media. Lipid development in mature (white arrow) and immature adipocytes (yellow arrow) was assessed following Oil Red O staining. (b) NOD derived MSCs at early, mid and late passage number were cultured *in vitro* with control or osteogenic differentiation media. Calcium development (white arrows) was assessed with Alizarin Red staining. (c) NOD derived MSCs at early, mid and late passage number were cultured *in vitro* with control or chondrogenic differentiation media for 18 days. Filamentous glycosaminoglycan development during chondrogenesis (indicated by white arrows) was assessed with Alcian blue staining. Images were acquired on a Leica DM microscope, 20x magnification, scale bar = 100µm.

Supplementary Table 1: List of oligonucleotide primers for RT-PCR

Gene target	Forward primer	Reverse primer	Annealing Temp (^{oC})	Cycle number	Product size (bp)	Existing/Designed
Foxa2	GAGCAGCGGCCAGCGAGTTA	CCCAGGCCGGCGTTCATGTT	61	40	131	Existing
EndogenousPdx 1	CCTTCGGGCCTTAGCGTGTC	CGCCTGCTGGTCCGTATTG	61	40	392	Existing
Endogenous Neurod1	GCTCCAGGGTTATGAGATCG	CTCTGCATTCATGGCTTCAA	53.7	40	204	Existing
Ins1	GACCAGCTATAATCAGAGACC	AGTTGCAGTAGTTCTCCAGCTG	56.8	30	368	Designed
Ins2	GAGTCCCACCCCACCCAG	TCCACTTCACGGCGGGACA	62	30	124	Existing
Gcg	ATGAAGACCATTTACTTTGTGGC TG	CGGCCTTTCACCAGCCACGC	60	35	380	Designed
Sst	ATGCTGTCCTGCCGTCTCCA	CTAACAGGATGTGAATGTCTTC CA	60	35	351	Designed
Ppy	TACTGCTGCCTCTCCCTGTT	CCAGGAAGTCCACCTGTGTT	61.2	35	268	Designed
Syp	ACCTCGGTGGTGTTTGGCTTCC	CCTGGAGGTGCGCGCATGAA	56.2	40	106	Existing
Nkx6.1	ATCTTCTGGCCCGGAGTGATG	GGCTGCGTGCTTCTTTCTCCA	62	40	284	Existing
Pcsk1	GATGGCTACACAGACAGCAT	GGATCAGCCAAATCCACCAG	52.9	40	406	Existing
Pcsk2	TCGCCAAGTTGCAGCCAGAAC	CTTCGGCCACGTTCAAGTCTA	52.9	40	313	Existing
Slc2a2	GGATAAATTCGCCTGGATGA	TTCCTTTGGTTTCTGGAACT	55	40	298	Existing
Gck	TATGAAGACCGCCAATGTGA	TTTCCGCCAATGATCTTTTC	55	40	244	Existing
Actb	CCATCATGAAGTGTGACGTTG	TACTCCTGCTTGCTGATCCA	61	30	243	Existing
Exogenous Neurod1	TCAACCCTCGGACTTTCTTG	ATGTTGTGGCGGATCTTGAAG (binds eGFP)	61	30	1081	Existing
Exogenous Pdx1	CCCCCACAGTTCACATCTAGC	CTCTGTGCCGTTAGGGAATG	62	30	180	Designed

Supplementary Table 2A: Average stained cell acquisition and analysis of NOD BMSCs and double positive cells

Cell staining sample	All events (%)	Viable cells (%)	Singlets (%)	APC events (%)	PE events (%)	CD45⁺/Ly6⁺ events (%)	CD45⁻/Ly6⁺ events (%)
**Unstained**	20,000	18,270	16,781	-	-	-	-
	100	91.35 ± 6.57%	83.91 ± 13.24%				
**CD45-APC**	20,000	18,498	17,144	11,781	-	-	-
	100	92.49 ± 5.32%	85.72 ± 11.15%	58.91 ± 15.32%			
**Ly6-PE**	20,000	18416	16272	-	11675	-	5274
	100	92.08 ± 4.78%	81.36 ± 9.67%		58.38 ± 12.87%		26.37 ± 24.17%
**CD45-APC/Ly6-PE**	10,000	14245	13189	-	-	2547	3191
	100	94.96 ± 3.03%	87.93 ± 7.80%			16.98 ± 0.26%	21.27 ± 8.96%

Footnotes: Stromal cells were divided into four groups for FACS analysis: Unstained, CD45-APC stained, Ly6-PE stained and CD45-APC/Ly6-PE stained. Two sub-populations of cells were sorted from stromal cells stained with both CD45-APC and Ly6-PE: BMSC (orange) and double positive cells (red). The final percentages of each sorted cell population were determined as a fraction of the total cell count (n=2).

Supplementary Table 2B: Average purity analysis of sorted BMSCs and double positive cells

Sorted sample	All events (%)	Viable cells (%)	Singlets (%)	$CD45^+/Ly6^+$ events (%)	$CD45^-/Ly6^+$ events (%)
**Double positive ($CD45\text{-}APC^+/Ly6\text{-}PE^+$)**	498	299	292	83.4 ± 1.2%	5.25 ± 4.05%
	100	66.04 ± 16.58%	64.7 ± 16.69%		
**BMSC ($CD45\text{-}APC^-/Ly6\text{-}PE^+$)**	396.5	335.5	324.5	0.9 ± 0.9%	93.95 ± 0.95%
	100	85.18 ± 2.83%	82.69 ± 4.11%		

Footnotes: FACS sorted BMSCs (orange) and double positive cells (red) were further analysed for their purity with respect to CD45-APC and Ly6-PE marker expression. The final percentages for purity of each sorted cell population were determined as a fraction of the total cell count (n=2).

Supplementary Table 3A: Persistence of bioluminescent signal in NOD/*Scid* mice

	Week												
	0	1	2	3	4	5	6	7	8	9	10	11	12
**$1x10^4$ cells**	****	***	ns	ns	ns	ns	ns	ns	ns	ns	ns	ns	ns
**$1x10^5$ cells**	****	****	****	***	***	***	***	***	***	**	ns	*	ns
**$1x10^6$ cells**	****	****	****	****	****	***	****	***	***	***	**	**	*

Footnotes: A two-way ANOVA with Sidak's post-hoc was performed comparing BLI of animals transplanted with tracking BMSCs in comparison to normal animals for the duration of the experiment. Data is represented as significance, * p<0.05.

Supplementary Table 3B: Persistence of bioluminescent signal in NOD mice

	Week			
	0	1	2	3
**$1x10^4$ cells**	****	ns	ns	ns
**$1x10^5$ cells**	****	*	ns	ns
**$1x10^6$ cells**	****	****	*	ns

Footnotes: A two-way ANOVA with Sidak's post-hoc was performed comparing BLI of animals transplanted with tracking BMSCs in comparison to normal animals for the duration of the experiment. Data is represented as significance, * p<0.05.

Supplementary Table 4A: Cell acquisition analysis of lentivirus transduced BMSCs

Transduction	All events (%)	Viable cells (%)	Singlets (%)	GFP⁺ (%)	mCherry⁺ (%)	GFP⁺/mCherry⁺ (%)
**Untransduced**	10,000	8,996	8,755	15	-	-
	100	90.0	87.6	0.0		
**HMD**	10,000	4,732	3,723	788	-	-
	100	47.3	37.2	7.9		
**HMD-*INS-FUR***	10,000	7,582	7,123	5,995	-	-
	100	75.8	71.2	60.0		
**HMD-*NeuroD1***	10,000	8,832	5,854	1,149	-	-
	100	88.3	58.5	11.5		
**HMD-*INS-FUR/NeuroD1***	10,000	6,081	5,297	2,465	-	-
	100	60.8	53.0	24.7		
**HMD-*Pdx1***	10,000	8,623	8,568	17	3,081	28
	100	86.2	99.4	0.0	36.0	0.0
**HMD-*INS-FUR* & HMD-*Pdx1***	10,000	7,444	7,384	3,559	2,245	1,500
	100	74.4	99.2	48.2	30.4	20.3

Transduced BMSCs were divided into seven groups for FACS analysis: Untransduced BMSC-*Luc2*, BMSC-*Luc2* transduced with HMD, HMD-*INS-FUR*, HMD-*NeuroD1*, HMD-*INS-FUR/NeuroD1*, HMD-*Pdx1,* and HMD-INS-FUR followed by HMD-*Pdx1*. Transduced BMSCs were sorted dependent on their fluorescent profiles as GFP^+ (green), $mCherry^+$ (red) and $GFP^+/mCherry^+$ (yellow) respectively. The final percentages of each sorted cell population were determined as a fraction of the total cell count.

Supplementary Table 4B: Sorted cell purity analysis of lentivirus transduced BMSCs

Sorted cells	All events (%)	Viable cells (%)	Singlets (%)	GFP^+ (%)	$mCherry^+$ (%)	$GFP^+/mCherry^+$ (%)
**BMSC-*Luc2-HMD***	147	80	72	45	-	-
	100	54.4	49.0	30.6		
**BMSC-*Luc2-INS-FUR***	118	89	84	73	-	-
	100	75.4	71.2	49.7		
**BMSC-*Luc2-NeuroD1***	208	162	160	93	-	-
	100	77.9	76.9	44.7		
**BMSC-*Luc2-INS-FUR/NeuroD1***	119	87	81	68	-	-
	100	73.1	68.1	57.1		
**BMSC-*Luc2/CDUPRT-Pdx1***	1000	930	916	0	888	5
	100	93	98.5	0	97.0	0.5
**BMSC-*Luc2/CDUPRT-INS-FUR-Pdx1***	1014	955	941	43	801	834
	100	94.2	98.5	4.6	85.1	88.6

Footnotes: Transduced BMSCs were sorted in to six distinct GFP^+ (green), $mCherry^+$ (red) and $GFP^+/mCherry^+$ (yellow) cell populations via FACS analysis. The final purity percentages of each sorted cell population were determined as a fraction of the total cell count.

Supplementary Table 5A: Significance in blood glucose concentration following BMSC-*INS-FUR* transplant compared to normal controls

	Days																		
	0	1	2	3	4	5	6	7	8	9	10	11	12	13	14	15	16	17	18
**Diabetic**	ns	ns	ns	ns	ns	****	ns	****	****	****	***	****	****	****	****	****	****	****	****
**1x10^{7}**	ns	ns	****	****	****	****	****	****	****	****	****	****	****	****	****	****	****	****	****
**5x10^{7}**	ns	ns	*	****	****	****	***	****	****	*	**	****	****	**	**	**	ns	*	*
		19	20	21	22	23	24	25	26	27	28	29	30	31	32	33	34	35	36
**Diabetic**		****	****	****	****	****	-	-	-	-	-	-	-	-	-	-	-	-	-
**1x10^{7}**		****	****	****	****	****	****	****	****	****	****	****	****	****	****	****	****	****	****
**5x10^{7}**		**	ns	**	**	***	***	****	****	***	****	****	****	**	ns	****	***	***	****

Footnotes: A two-way ANOVA with Sidak's post-hoc was performed comparing blood glucose concentrations of diabetic and treated animals in comparison to normal animals for the duration of the experiment. Data is represented as non-significant (ns) or significant, * $p<0.05$.

Supplementary Table 5B: Significance in body weight following BMSC-*INS-FUR* transplant compared to normal controls

	Days																		
	0	1	2	3	4	5	6	7	8	9	10	11	12	13	14	15	16	17	18
**Diabetic**	ns	ns	ns	*	ns	*	***	ns	*	**	***	*	ns	ns	*	ns	ns	ns	ns
**1x10^{7}**	***	*	**	***	***	***	****	***	***	***	****	**	**	*	**	*	*	*	ns
**5x10^{7}**	****	****	****	****	****	****	****	****	****	****	****	****	****	***	****	**	***	***	*
		19	20	21	22	23	24	25	26	27	28	29	30	31	32	33	34	35	36
**Diabetic**		ns	*	ns	ns	ns	-	-	-	-	-	-	-	-	-	-	-	-	-
**1x10^{7}**		**	**	**	***	**	*	ns	ns	ns	ns	ns	ns	ns	ns	ns	ns	ns	ns
**5x10^{7}**		***	****	****	****	****	****	**	****	****	****	***	**	****	**	**	*	ns	ns

Footnotes: A two-way ANOVA with Sidak's post-hoc was performed comparing body weights of diabetic and treated animals in comparison to normal animals for the duration of the experiment. Data is represented as non-significant (ns) or significant, * $p<0.05$.
